# Evaluating the Educational Impact of Medical Interdisciplinary Research and Academic Collaboration for Excellence (MIRACLE) 2025: Insights From an Undergraduate Medical Conference at Madras Medical College

**DOI:** 10.7759/cureus.98004

**Published:** 2025-11-28

**Authors:** Yogesh S, Kavitha M, KR Sethuraman, Sunil Kumar S, Vishal Rajkumar, Sahasyaa Adalarasan

**Affiliations:** 1 Internal Medicine, Madras Medical College and Rajiv Gandhi Government General Hospital, Chennai, IND; 2 Master of Health Professions Education (MHPE), Institute of Health Professions Education, Sri Balaji Vidyapeeth, Puducherry, IND; 3 Microbiology, Madras Medical College, Chennai, IND; 4 Medicine, Asian Institute of Medicine, Science and Technology (AIMST) University, Bedong, MYS; 5 Internal Medicine, Madras Medical College, Chennai, IND

**Keywords:** conference, madras medical college, medical conference, medical education, miracle

## Abstract

Background

Undergraduate medical conferences provide a valuable yet often underutilized platform for fostering clinical reasoning, research aptitude, and interdisciplinary learning. Medical Interdisciplinary Research and Academic Collaboration for Excellence (MIRACLE) 2025, conducted at Madras Medical College, was the institution’s first full-scale undergraduate conference aimed at integrating academic learning with practical exposure.

Methods

Feedback from participants was analyzed to assess the educational impact of the conference. The medicine workshop was selected for detailed evaluation, with 40 participants attending and 24 submitting feedback through a structured Google Form. The questionnaire used a Likert-type scale to assess five speaker-related parameters and six overall domains. Additionally, four plenary sessions held in the general auditorium were evaluated by 221, 222, 205, and 245 participants, respectively. Quantitative data were analyzed descriptively, and open-ended responses underwent thematic analysis.

Results

The medicine workshop received overwhelmingly positive feedback, with 16-19 participants on average rating each parameter as “done well.” High scores were reported for content coverage, interactivity, and audiovisual quality, while time management received comparatively lower ratings. Thematic analysis revealed appreciation for the clarity and approachability of faculty, along with requests for longer, more hands-on sessions. Similarly, plenary sessions were well-received, with the majority of respondents awarding scores of 5 across both days.

Conclusion

MIRACLE 2025 successfully demonstrated the educational potential of undergraduate conferences. Participant feedback reflected high satisfaction with both the scientific and interactive components, highlighting the role of such events in bridging theoretical learning and clinical practice. Structured feedback and future objective assessments could further strengthen the impact of similar initiatives in medical education.

## Introduction

Medical education serves as the cornerstone of every health decision that healthcare workers make. Proper medical education is of high importance, considering the ever-growing need for competent physicians. Medical education, along with the education of attitude, ethics, leadership skills, and communication, has taken the forefront [1]. The current competency-based medical education (CBME) model has been widely implemented across Indian medical colleges, aiming to provide such comprehensive training [2]. Conferences are also an excellent way of delivering medical education [3]. However, these conferences often seem to be limited to postgraduates and professors. Such active, learner-centered formats can enhance students’ education compared to traditional lecture-based methods [4].

Medical Interdisciplinary Research and Academic Collaboration for Excellence (MIRACLE) 2025 was a celebration of the spirit of medical education and inquiry. Designed as the first-ever full-scale undergraduate conference in India, it was spread across three days (April 30, 2025 - January 2, 2025). It offered an inclusive space for budding medical professionals to engage with advanced concepts, practical skill-building, and interdisciplinary approaches to patient care. The event's structured scientific sessions, hands-on workshops, and interactive discussions covered a wide spectrum of clinical specialties, ranging from general surgery and neurology to pediatrics, forensic medicine, and rehabilitation. With opportunities for poster presentations, case-based learning, and expert lectures from renowned professionals across India, participants were encouraged to translate textbook knowledge into bedside practice (Appendix 1).

What set MIRACLE apart from other academic conferences was its strong interdisciplinary focus. Each topic was explored through the perspectives of experts from diverse specialties across the globe, ensuring a comprehensive and multifaceted understanding. The case-based discussions, featuring both common and rare real-world scenarios, further enriched the learning experience by emphasizing clinical relevance and practical decision-making.

Beyond academic rigor, the conference also fostered creativity, ethical awareness, and leadership among participants. Competitions such as Photo-Voice, Paint Your Thought, and Game-Based Learning encouraged attendees to engage with the humanistic, artistic, and technological dimensions of medicine. Dedicated sessions on artificial intelligence, legal considerations in clinical practice, and strategies for personal and professional wellness provided valuable tools for navigating the evolving landscape of modern healthcare. The event concluded with concept culturals, which added an enjoyable, interactive element to the program and demonstrated that learning can be both meaningful and fun.

Such unique methods have been proven to spark interest and create a more significant impact on students [5]. With the support of eminent faculty, government officials, and healthcare leaders, MIRACLE nurtured a generation of empathetic, innovative, and well-rounded medical professionals who were prepared not only to treat diseases but also to advance medical knowledge and patient care. With several registrations made within hours of commencement, MIRACLE proved to be a game changer in the new era of medicine.

## Materials and methods

Study design and setting

This study adopted a cross-sectional descriptive design aimed at assessing participant perceptions of MIRACLE 2025, the first full-scale undergraduate medical conference held at Madras Medical College (MMC), Chennai. Chennai, one of India’s major metropolitan cities, hosts a diverse medical education environment with multiple government and private institutions. MMC, established in 1835, is one of the oldest and most reputed medical schools in South Asia and is affiliated with the Dr. M.G.R. Medical University, Tamil Nadu. The college has an annual intake of 250 undergraduate MBBS students, representing a mix of urban and semi-urban populations from across the state. The grand sum of interactive case-based discussions (concrete experience), facilitated reflection and feedback (reflective observation), and faculty-guided conceptual clarification (abstract conceptualization), culminating in hands-on skill demonstrations and clinical reasoning exercises (active experimentation), provided a unique intervention aimed at enhancing student learning.

Study population and inclusion criteria

The study population comprised undergraduate medical students from multiple medical colleges across India who attended MIRACLE 2025. The event attracted participants representing a broad spectrum of geographical and institutional backgrounds, reflecting the diversity of India’s medical education community. For the present analysis, feedback from the medicine workshop and the general auditorium sessions was included.

All undergraduate students registered for the medicine workshop were eligible to participate. A total of 40 students attended, representing mixed academic years and varied regions across India. All 40 participants attended the workshop in full; however, only 24 students completed the feedback form circulated online later that evening, forming the final sample for analysis. Participants who did not submit the online form or provided incomplete responses were excluded.

Feedback from the general auditorium sessions was collected separately. These sessions were open to all conference registrants and held on two consecutive days, with four plenary sessions (Day 1 forenoon and afternoon; Day 2 forenoon and afternoon). Average attendance ranged from 205 to 245 participants per session, representing a broader undergraduate cohort that included attendees from other workshops. The sessions featured eminent national and international speakers on diverse, clinically relevant topics such as Liver Transplantation: A Clinical Perspective (Dr. Mohamed Rela, Chennai), Artificial Intelligence in Medical Education (Dr. Avinash Supe, Mumbai), and The Intersection of Medicine and Law: A Judge’s Perspective on Medicolegal Challenges in Clinical Practice (Hon’ble Justice Dr. G. Jayachandran). All talks were exclusive to MIRACLE 2025 and curated to reflect cross-disciplinary themes in modern medicine.

Institutional Ethics Committee (IEC) approval was obtained from Madras Medical College under the category of educational research exemption, as the study involved anonymous, voluntary student feedback without any intervention or identifiable data.

Data collection procedure

Feedback for the medicine workshop was obtained through a structured Google Form distributed via WhatsApp at the end of the session. Responses were collected within 24 hours. The questionnaire used a performance-based Likert-type scale (rather than an agreement-based scale) because the intent was to evaluate teaching demonstration rather than attitudinal agreement. The categories, done well, moderate extent, to some extent, not demonstrated, and could not assess, were adapted from the Teaching Evaluation Framework commonly used for faculty feedback in Indian medical institutions (Appendix 2). Each participant rated all ten speakers across five parameters: referring adequately to the content/topic; presenting and discussing relevant concepts; explaining concepts with suitable examples; engaging participants during the session; and demonstrating the ability to raise or answer queries.

Additionally, participants rated the overall session on six broader domains, content coverage, concept attainment, interactivity, relevance to work, audiovisual quality, and time management, using a 5-point Likert scale (1 = Poor to 5 = Excellent). The inclusion of both scales enabled a comprehensive assessment: the first captured teaching performance, while the second reflected the overall learning experience. An open-ended question (“Were there any specific comments you wanted to tell us?”) was also included to capture qualitative perceptions.

Feedback for the general auditorium sessions followed the same 5-point rating system (1 = Poor to 5 = Excellent). The total number of respondents for the four sessions were 221, 222, 205, and 245, respectively. Each session’s feedback link was distributed immediately following the session’s conclusion to ensure that only complete attendees provided responses.

Data analysis

All responses were anonymized before analysis to ensure confidentiality, and randomization of entries was performed to minimize identification bias. Quantitative data obtained from the Likert-type items were analyzed using descriptive statistics, including frequencies and mean values, to provide a spectrum-based overview of participant experiences [6].

Open-ended qualitative responses were analyzed through thematic analysis using an inductive, manual approach. Responses were first read repeatedly to ensure familiarization with the content. Descriptive codes were then assigned to recurring words or phrases that reflected participants’ opinions or experiences. Related codes were grouped to form preliminary categories, which were further refined into overarching themes representing positive perceptions, suggestions for improvement, and logistical or organizational feedback. Themes were reviewed and consolidated through consensus among the authors to ensure consistency and credibility.

This combined approach of descriptive quantitative and inductive qualitative analysis allowed for a comprehensive interpretation of participant feedback, capturing both measurable outcomes and experiential nuances [7].

## Results

Medicine workshop

**Figure 1:**
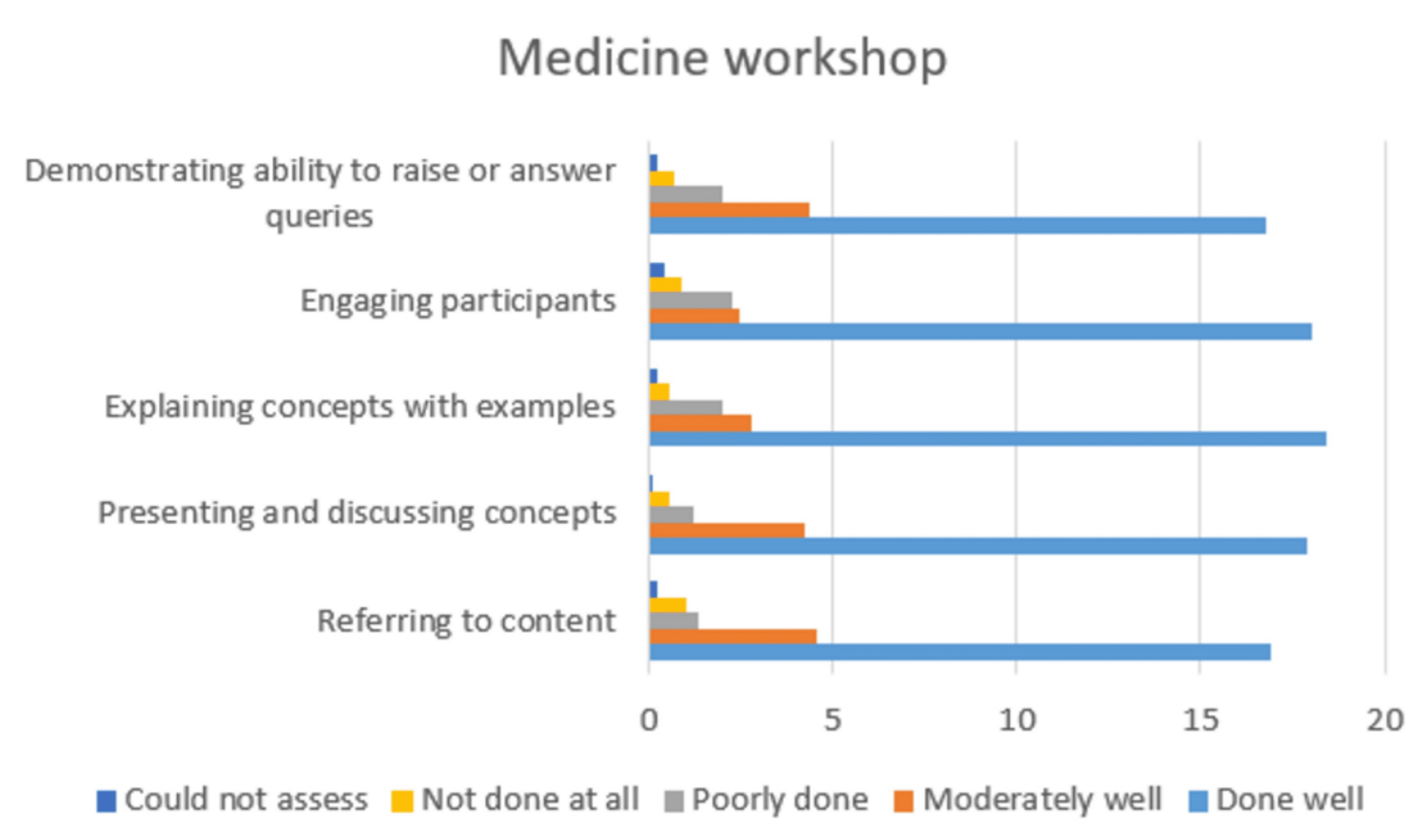
Medicine workshop feedback.

Of the 40 students who attended the medicine workshop, 24 submitted feedback through the online questionnaire. Analysis of speaker-specific ratings showed consistently high scores across all five parameters. On average, 16-19 respondents per speaker rated “done well,” while 2-4 rated “moderately well” (Table 1). Only a small minority (typically 1-2 respondents) marked “to some extent” or “not demonstrated,” and “could not assess” was rarely selected (Figure 1). These results indicate that the majority of participants perceived the workshop as well structured and effectively delivered, with only isolated concerns regarding depth or coverage.

**Table 1:** Feedback from the medicine workshop.

Rating (Likert scale)	Referring to content (mean)	Presenting and discussing concepts (mean)	Explaining concepts with examples (mean)	Engaging participants (mean)	Demonstrating ability to raise or answer queries (mean)
Done well	16.89	17.89	18.44	18	16.78
Moderately well	4.56	4.22	2.78	2.44	4.33
Poorly done	1.33	1.22	2	2.23	2
Not done at all	1	0.56	0.56	0.89	0.67
Could not assess	0.22	0.11	0.22	0.44	0.22

When asked to rate the overall workshop across six domains, most participants awarded the highest score (5 = excellent) for content coverage, concept attainment, interactivity, relevance to work, and audiovisual quality. Time management received comparatively lower ratings, with a higher proportion of responses in the “moderate” and “good” categories.

Analysis of open-ended responses revealed three broad themes: positive learning experiences, suggestions for improvement, and logistical considerations. Participants described the sessions as “fascinating,” “clinically enriching,” and “inspiring,” frequently emphasizing the clarity, approachability, and humility of faculty members. Many noted that the workshop’s interactive and case-based format helped them translate theoretical knowledge into clinical understanding, deepening their interest in medicine and enhancing motivation toward patient-centered learning.

Constructive feedback primarily focused on time constraints, with several participants suggesting longer or repeated sessions and a greater emphasis on hands-on activities and structured discussions. A smaller proportion mentioned logistical challenges such as minor scheduling overlaps and venue limitations, though these did not significantly affect the overall learning experience. Collectively, the qualitative findings reinforced the quantitative results, demonstrating that the medicine workshop was perceived as a highly effective and engaging educational intervention.

General auditorium sessions

Feedback for the four general auditorium sessions revealed strong overall satisfaction (Table 2). For the Day 1 forenoon session (n = 221), the majority awarded the highest score (180 rated 5, 31 rated 4), with only a small number assigning scores of 3 (n = 6), 2 (n = 2), or 1 (n = 2). The Day 1 afternoon session (n = 222) showed a similar trend, with 179 participants rating it 5, 32 rating it 4, 10 rating it 3, and only 1 rating it 2.

**Table 2:** General auditorium sessions' feedback.

Rating (Likert scale)	Day 1 (Forenoon)	Day 1 (Afternoon)	Day 2 (Forenoon)	Day 2 (Afternoon)
5	180	186	171	195
4	31	27	20	36
3	6	5	0	8
2	2	1	4	5
1	2	3	10	1

**Figure 2:**
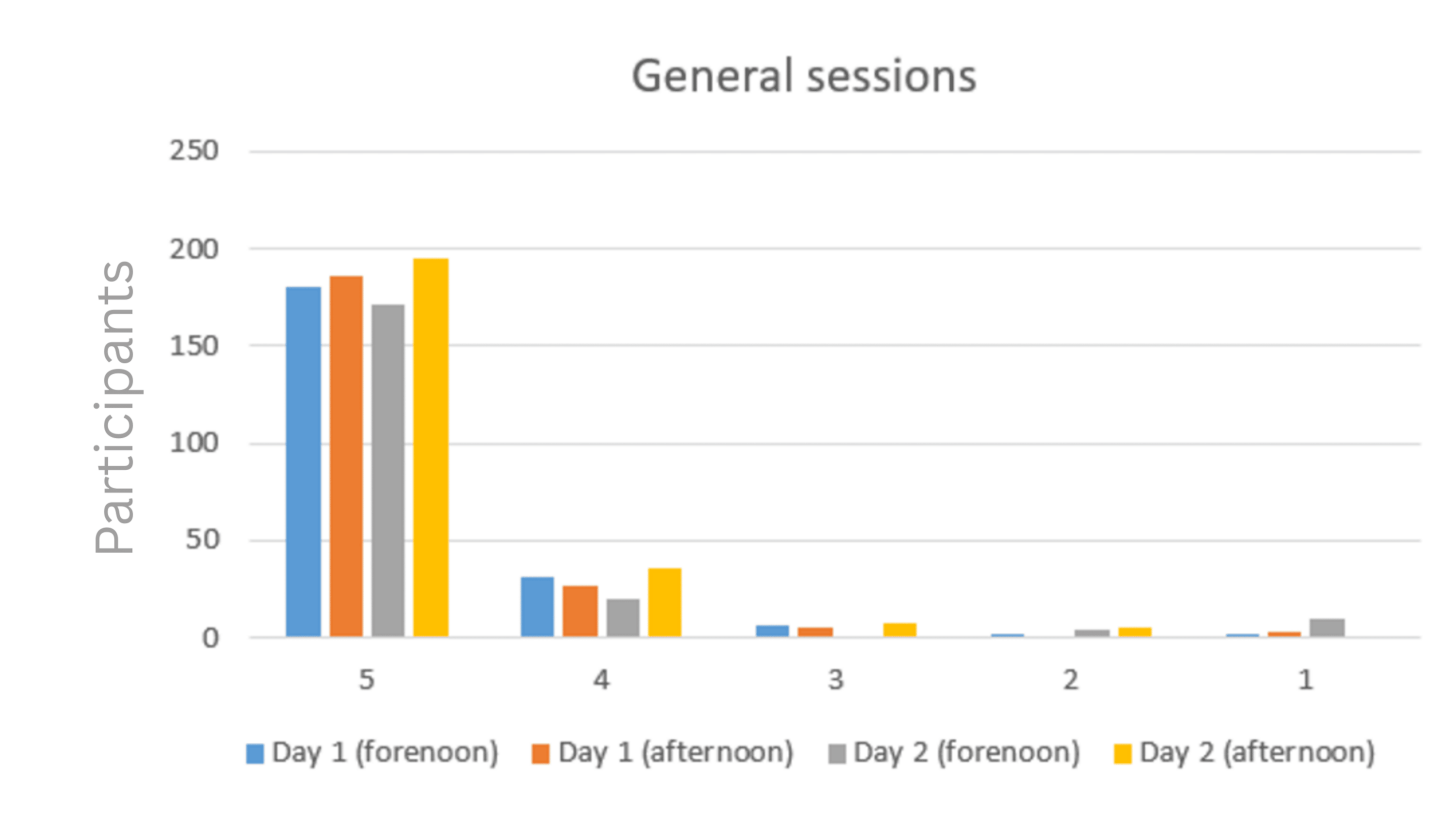
General auditorium sessions' feedback.

The Day 2 forenoon session (n = 205) had 171 participants rating it 5, 20 rating it 4, none rating it 3, 4 rating it 2, and 10 rating it 1, reflecting a slightly wider spread of responses but still a predominantly positive outlook. The Day 2 afternoon session (n = 245) had the strongest feedback, with 195 participants rating it 5, 36 rating it 4, 8 rating it 3, 2 rating it 2, and 1 rating it 1 (Figure 2).

Taken together, these results suggest that the plenary sessions were well received by the majority of attendees, with consistently high ratings across both days. The feedback patterns indicate strong satisfaction with content delivery and engagement, although occasional lower ratings suggest variability in individual session appeal.

## Discussion

Conferences and workshops have long been recognized as valuable platforms for the dissemination of medical knowledge, skill enhancement, and professional networking [8]. However, undergraduate medical students are rarely given opportunities to participate in or contribute to such academic forums, which are often tailored for postgraduate trainees or faculty members. This lack of exposure limits early engagement with research, critical thinking, and interdisciplinary learning, skills that are essential in shaping well-rounded clinicians. Such exposure has also been proven to have long-term benefits [9].

In this context, MIRACLE 2025 represents a landmark initiative. As the first full-scale undergraduate conference organized at Madras Medical College, it successfully integrated lectures, workshops, competitions, and interactive discussions tailored to the undergraduate level. The medicine workshop, chosen for evaluation, demonstrated how structured clinical teaching, coupled with active faculty involvement, can stimulate curiosity and strengthen core competencies. The overwhelmingly positive feedback reflects not only the enthusiasm of participants but also the potential of such platforms to reshape undergraduate medical education in India. Networking also plays a major role in such conferences, as described in the literature [10].

Many similar evaluations of new teaching methods have been conducted in the past. A study on a nuclear medicine training program revealed that novel teaching methods received better reception from trainees [11]. A similar study conducted for a faculty-training program concluded in a comparable manner [12]. Another study conducted during the COVID-19 pandemic at the Arabian Gulf University was representative of the Middle Eastern population [13]. However, no large-scale studies have been conducted in the Indian population evaluating unique teaching methods. Such studies, when conducted, could potentially influence policies and teaching methodologies [14,15].

The present evaluation also revealed challenges. A significant proportion of participants (16 out of 40) did not provide feedback, limiting the generalizability of the results. Moreover, the reliance on self-reported perceptions rather than objective assessments of knowledge or skill acquisition introduces an element of subjectivity. Future evaluations could incorporate structured pre- and post-tests, larger sample sizes, and mixed-methods approaches to strengthen the evidence on educational impact. Despite these limitations, this initiative highlights the feasibility and value of engaging undergraduates in conference-style learning, an area that has remained underexplored in the Indian medical education system.

## Conclusions

The evaluation of the medicine workshop at MIRACLE 2025 underscores the importance of creating structured, engaging, and student-centered platforms for undergraduate medical education. Despite certain limitations, the overwhelmingly positive response demonstrates that such initiatives can foster deeper clinical understanding, encourage active participation, and inspire professional growth.

Expanding and refining undergraduate-focused conferences may help bridge the gap between classroom teaching and real-world medical practice, ultimately contributing to the development of more competent and motivated physicians.

## Figures and Tables

**Video 1:** Brochure of MIRACLE 2025. MIRACLE: Medical Interdisciplinary Research and Academic Collaboration for Excellence.

**Figure 3:**
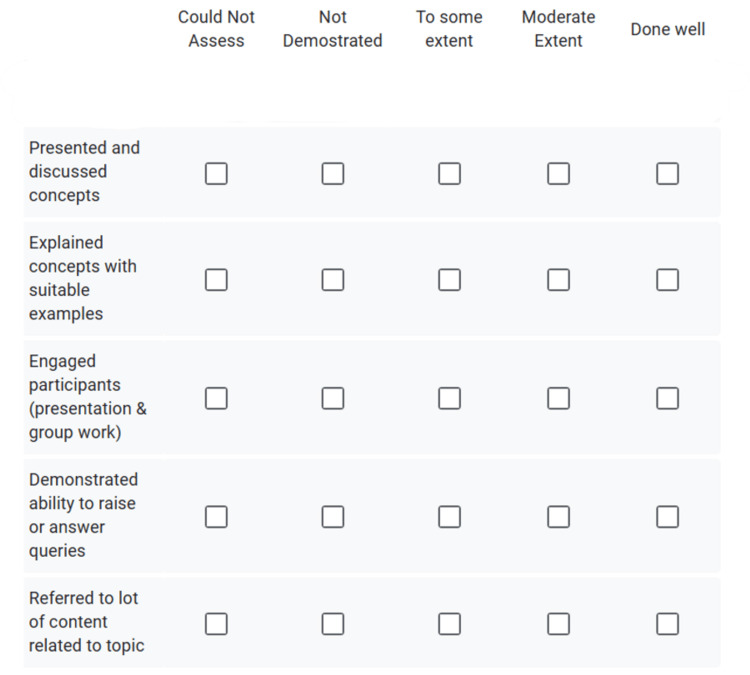
Questionnaire.

## Appendices

Appendix 1

Appendix 2
